# Gastric Cancer Stem Cells: Current Insights into the Immune Microenvironment and Therapeutic Targets

**DOI:** 10.3390/biomedicines8010007

**Published:** 2020-01-06

**Authors:** Lingfeng Fu, Luke Bu, Tadahito Yasuda, Mayu Koiwa, Takahiko Akiyama, Tomoyuki Uchihara, Hideo Baba, Takatsugu Ishimoto

**Affiliations:** 1 Gastrointestinal Cancer Biology, International Research Center of Medical Sciences (IRCMS), Kumamoto University, Kumamoto 860-0811, Japan; flf_0228@yahoo.co.jp (L.F.); buluke1221@yahoo.co.jp (L.B.); kumasyuwa@gmail.com (T.Y.); mk.transit.in@gmail.com (M.K.); theaki50220@gmail.com (T.A.); tomoyuki.u.mikawa1@gmail.com (T.U.); 2 Department of Gastroenterological Surgery, Graduate School of Medical Sciences, Kumamoto University, 1-1-1 Honjo, Kumamoto 860-8556, Japan

**Keywords:** gastric cancer, gastric cancer stem cell, immune microenvironment, cancer therapy

## Abstract

Gastric cancer (GC) is a leading cause of cancer-related death worldwide. Cancer stem cells (CSCs) are known to be involved in chemotherapy resistance and the development of metastases. Although CSCs harbor self-renewal and tumorigenic abilities, the immune microenvironment surrounding CSCs provides various factors and supports the maintenance of CSC properties. The current review summarizes the accumulating findings regarding the relationship between the immune microenvironment and gastric CSCs (GCSCs), which will support the possibility of developing novel therapeutic strategies for targeting GCSCs.

## 1. Introduction

Gastric cancer (GC) is fifth in incidence and third in mortality among all cancers worldwide (GLOBOCAN 2018). In epidemiological studies, the incidence of GCs is generally approximately twice as high in men as in women, and there are great differences among different countries [1]. Eastern Asia, Central and Eastern Europe, and South America have higher incidence rates than Northern America and most parts of Africa [1]. These regional differences reflect differences in dietary patterns, food storage, and fresh produce availability, as well as differences in the prevalence of *Helicobacter pylori* infection [2].

Despite advances in treatment for GC patients, tumor relapse and metastasis lead to poor overall survival. The cancer stem cell (CSC) model has been proposed to explain tumor relapse and resistance [3]. CSCs have been identified in many solid malignancies, including GCs, and targeting the CSC population may be essential to prevent tumor relapse and spread [4]. In addition, specific markers of CSCs have been explored in recent decades. A large number of studies have shown that CSC tends to share cell surface markers with tissue stem cells, and the expression of CSC markers will affect the characteristics of CSC, including tumorigenicity, chemoresistance and invasive abilities [5]. Because of this, it also provides guidance for investigations on CSC markers.

This review provides a better understanding of the role of gastric cancer stem cells (GCSCs) in GC progression and the plasticity mediated by the tumor microenvironment.

## 2. GCSC Markers

CD44 was the first GCSC marker identified, and it was found by using GC cell lines. The CD44 positive cells have obvious tumorigenic characteristics. It is known that CD44 positive cells do not only form spheroids in vitro, but also form tumors when injected into the gastric wall of immunodeficient mice [6]. In addition, CD44 positive/CD24 positive cells are also found as CSCs in GC tissues. An investigation further showed that the CD44 positive /CD24 positive fraction demonstrated higher tumorigenicity than the CD44 negative/CD24 negative fraction when injected into immunodeficient mice. CD44 positive /CD24 positive cells have been suggested to have the ability of self-renew and to produce differentiated progeny as CSCs, suggesting that the combined expression of CD44 and CD24 can be used as a possible GCSC marker [7]. In addition, the cell-surface markers CD44 and CD54 can be used to isolate CSCs from the peripheral blood of GC patients, and tumors generated by CD44 positive /CD54 positive cell transplantation into the immunodeficient mice are similar to the original tumors in patients. CD44 positive/CD54 positive cells are identified as markers of GCSCs because these cells can differentiate into gastric epithelial cells in vitro and these kind of cells have the ability to undergo self-renewal in vivo. [8]. Similarly, the combination of epithelial cell adhesion molecule (EpCAM) and CD44 have also been found as putative GCSC markers. The EpCAM positive/CD44 positive fraction in human GC tissues has the tumorigenic ability after injection into immunodeficient mice, maintains histological differentiation, and reproduce the phenotypical heterogeneities of the primary tumors. In addition, this fraction has a stronger resistance to anticancer drugs than the other fractions [9].

Aldehyde dehydrogenase 1 (ALDH1) has been used as a marker for cancer-initiating cells (CICs), and ALDH1 positive cells have been detected in diffuse GC in recent years; this is because ALDH1 positive cells show strong tumorigenicity, self-renewal and the ability to generate tumor hierarchy and heterogeneity in vivo. ALDH1 positive cells are also one of the markers of GCSCs. Further studies have shown that ALDH1 positive GCSCs are involved in regenerating islet-derived family member 4 (REG4), which is a factor related to tumorigenicity, cell growth, survival and apoptosis. The REG4 expression is down-regulated by transforming growth factor-β (TGF-β) in ALDH1 positive GCSCs, which correlates with reductions in the GCSC population and tumorigenicity [10,11].

Various studies have investigated whether GCSCs are enriched through spheroid formation in a human GC cell line in defined serum-free medium. Spheroid body-forming cells are recognized to have GCSC properties, including self-renewal, continuous proliferation, drug resistance, high tumorigenicity, and over-expression of CD44 and other stem cell related genes and proteins [12]. Another group demonstrated that CD90 might be a potential GCSC marker. CD90 positive GC cells showed a greater tumorigenic ability in vivo than CD90 negative GC cells and could reestablish the hierarchical tumors from a single tumor cell, demonstrating their self-renewal properties. In addition, ERBB2 was highly expressed in about 25% of gastric tumor models, which correlated with the elevated level of CD90 expression in these tumors. Treatment with trastuzumab could reduce the CD90 positive GCSC frequency in the whole tumor mass and suppress tumor growth when combined with conventional chemotherapeutic agents [13].

The CD71 negative population is enriched in MKN1 cells after treatment with 5-fluorouracil and accumulates during the G0/G1 cell cycle phase. The CD71 negative population shows high resistance to conventional chemotherapeutic agents, which indicates their stem-like cell properties. Additionally, serial transplantation assays have demonstrated that the CD71 negative population has higher tumorigenicity than the CD71 positive population [14]. It has been proved that CD133 is a candidate molecule for GCSC markers. The expression of three candidates of CSC markers, ATP-binding cassette subfamily B member 1, ATP-binding cassette subfamily G member 2, and CD133, were investigated in 90 human GC tissue samples. The expression levels of these markers in GC varied with the histological differentiation status; poorly differentiated GC expressed these markers at a high level [15]. Moreover, the expression of CD133 in GC cells can be divided into two types: luminal expression in the gland and cytoplasmic expression. Notably, the cytoplasmic CD133 expression was useful as an independent prognostic factor in GC patients [16].

A few new markers have been identified in recent years. C-X-C chemokine receptor type 4 (CXCR4) is a CSC marker, and CXCR positive GCSCs can promote tumorigenicity in mice and chemoresistance due to their dormancy [17]. The adult stem cell marker leucine-rich repeat-containing, G protein-coupled receptor 5 (Lgr5) has been shown to be involved in the properties of CSCs in cancer cell populations. Moreover, Lgr5 is more highly expressed in a significant fraction of gastrointestinal tumors than in normal tissue [18,19]. Given this evidence, Lgr5 may be a possible GCSC marker. We summarize these markers in Table 1. Accumulating evidence suggests that GCSCs may exist at the base of GCs; thus, the understanding of GCSC markers will help us to target GCSC signaling pathways to regulate the self-renewal and differentiation processes of GCSCs.

## 3. GCSCs and the Immune Microenvironment

Cancer tissue consists of various stromal cells, such as fibroblasts, vascular endothelial cells, pericytes, bone marrow-derived cells and adipocytes [20]. Some evidence has shown that CSCs interact with their surroundings and stromal cells for adaptive advantage and survival [21].

For instance, stromal cells can support CSC development by undergoing various kinds of interactions and cell fusion to form hybrid tumor cells [22]. The microenvironment surrounding CSCs, called the “CSC niche”, is known to regulate CSC properties, which regulate CSC proliferation and differentiation [23]. Recently, accumulating evidence has indicated that immune cells in the tumor microenvironment have an impact on cancer progression. In fact, the number of tumor-infiltrating macrophages affects cancer progression and prognosis, and these macrophages are called tumor-associated macrophages (TAMs) [24]. TAMs have been reported to contribute to tumor growth, cell extravasation and antitumor immunity in various types of cancer [25,26,27,28]. A few studies have demonstrated that TAMs provide pivotal signals to promote CSC survival, self-renewal, maintenance, and migration. In turn, CSCs deliver tumor-promoting cues to TAMs that further enhance tumorigenesis [29]. Milk-fat globule-epidermal growth factor-VIII (MFG-E8), which has been identified as a growth factor involving phagocytosis, angiogenesis, and immune tolerance, was highly produced from TAMs and could regulate the ability of CSCs to promote tumorigenicity and anticancer drug resistance [30]. Another study demonstrated that the interaction between TAMs and GCSCs was mediated by the expression of some factors, such as monocyte chemoattractant-1 (MCP-1), VEGF, cyclooxygenase-2 (COX-2)/PGE-2, and IFN-γ [31]. We also investigated the interaction between TAMs and GCSC markers and delineated the molecular mechanism of CD44 induction that allowed infiltrated macrophages in the tumor microenvironment to contribute to redox adaptation through CD44 upregulation by miR-328 suppression [32].

Moreover, there is no doubt that cancer-associated fibroblasts (CAFs) have a critical role in maintaining the characteristics of TAMs, and the cell–cell interaction between CAFs and TAMs induces the recruitment and activation of each cell type [33,34]. CXCL12 derived from CAFs effectively attracts monocytes, and then the monocytes display the M2 macrophage phenotype, which supports tumor invasion and progression [35]. Based on this evidence, TAMs recruited by CAFs may promote GCSC properties through the upregulation of CSC marker expression. In addition, several compelling studies have shown that CAFs enhance CSC capabilities in GCSCs. CAFs significantly promote spheroid formation and the expression of CSC markers in GC cell lines, OCUM-12/side population (SP) cells and OCUM-2MD3/SP cells. The impacts of CAFs on GCSCs are remarkably blocked by TGF-β inhibitors but not by fibroblast growth factor receptor or cMet inhibition. These findings suggest that CAFs may enhance GCSC properties through TGF-β signaling [36]. Genome-wide DNA methylation and H3K27me3 analyses revealed that CAFs had diverse and distinct DNA methylation and H3K27me3 patterns. Moreover, the authors showed that loss of H3K27me3 in CAFs led to secretion of the cancer cell niche factors, and among these factors, WNT5A might promote the aggressive characteristics of GCSCs [37].

Immune checkpoint blockade is a current topic in cancer therapy. Because immune checkpoint pathways can maintain self-tolerance and limit collateral tissue damage during the antimicrobial immune response, cancer cells can evade immune damage by utilizing these pathways [38]. Nivolumab, a human monoclonal IgG4 antibody, can block the human programmed cell death-1 (PD-1) receptor. This antibody has been proven to be a treatment strategy for advanced cancer with significant clinical efficacy [39]. Based on clinical evidence, several studies have investigated the interaction between immune checkpoint-related molecules and CSCs.

For instance, programmed death-ligand 1 (PD-L1), which is highly expressed on the surface of CSCs, can inhibit T cell activity [40]. B7-H1 (also known as PD-L1)-positive GCSCs show a higher proliferative ability than B7-H1-negative GCSCs [41]. In contrast, CD4 positive T cells can differentiate into distinct subsets of T cells: suppressive regulatory T (Treg) cells and T helper 17 (Th17) cells. The functions of Th17 cells are contradictory due to the dual ability of these cells to promote or inhibit cancer [42]. A previous study showed that CSCs could affect the balance between Treg and Th17 subsets in several types of cancer by altering the cytokines in the tumor microenvironment [43]. However, Treg cells can promote the expansion of CSCs by secreting IL-17 in response to hypoxia [44]. Moreover, an investigation of Treg and Th17 cells revealed that STAT3 functioned as a critical transcription factor in Th17 differentiation and repressed the development of Treg cells [45]. In addition, STAT3 is highly expressed in GCSCs and has a role in stemness features and invasive properties [46,47]. Taken together, these findings suggest that STAT3 may be a key factor regulating GCSC properties and Th17/Treg cells. Although there is some evidence showing the effects of CSCs on Th17/Treg cells, the exact role of GCSCs in the balance between Treg and Th17 cells remains unclear.

**Figure 1:**
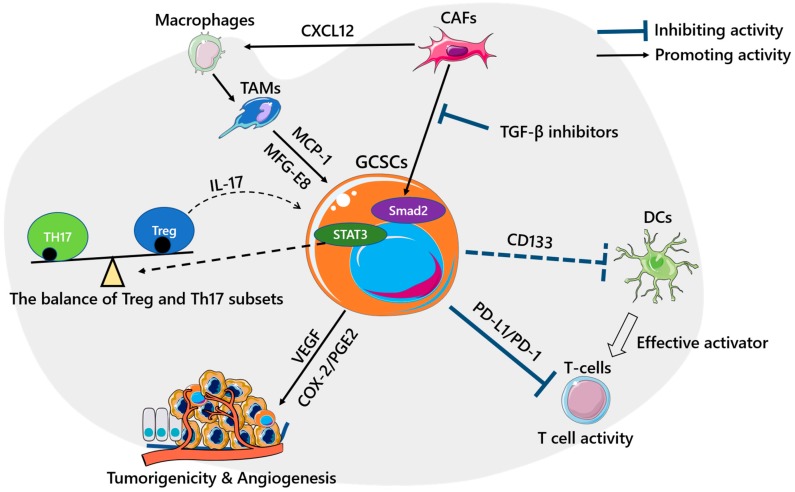
The roles of gastric cancer stem cells (GCSCs) in the immune microenvironment. Stromal cells can support GCSC development through various kinds of interactions. Tumor-associated macrophages (TAMs) enhance the induction of VEGF and COX2/PGE2 in GCSCs through monocyte chemoattractant-1 (MCP-1) and milk-fat globule-epidermal growth factor-VIII (MFG-E8). Cancer-associated fibroblasts (CAFs) not only directly enhance the CSC capabilities of GCSCs by activating the Smad2 pathway (TGF-β inhibitors inhibit this process) but also attract macrophages that are converted into TAMs by CXCL12. GCSCs can inhibit T cell activity through the PD-L1/PD-1 interaction. Some theories suggest that GCSCs affect the balance of Treg and Th17 subsets, and GCSCs impair the function of dendritic cells (DCs). The solid line represents strong evidences supporting theory in GCSC; the dashed line represents possible evidences guide theory in GCSC.

Dendritic cells (DCs) are professional antigen-presenting cells (APCs) and the most effective activators of resting or naive T cells in vitro and in vivo [48]. A recent study using CD133 positive, CD29 positive and CD44 positive CSCs showed that the number of activated DCs was decreased after CSC lysate/LPS (intracellular) or CSC conditioned medium/LPS (extracellular) stimulation. This result suggested that CSCs might impair the functions of DCs [49]. Although the relationship between DCs and GCSCs is still unclear, the functional DCs are required in the immune microenvironment for T cell activation. Thus, the relationship should be determined by further investigations (Figure 1).

Based on the fact that cancer tissues consist of various types of cells in addition to cancer cells, we need to understand the network formed by these cells. A better understanding of the relationship between GCSCs and the immune microenvironment will lead to the development of novel therapeutic strategies for advanced GCs.

## 4. Current Target and Future Direction of GCSC Treatment

Metastasis and recurrence are often detected in advanced GC patients after complete surgical resection, indicating that circulating tumor cells in the bloodstream or undetectable tumor nodules must exist at the time of surgery. Based on these experiences, there is a definitive consensus that compared with surgery alone, combination treatment strategies consisting of surgery with additional chemotherapy would improve the prognosis of GC patients [50]. However, conventional standard chemotherapy aimed at removing GC cells achieves limited benefits, which leads to a poor prognosis in advanced-stage GC patients. CSCs are thought to be enriched in the remaining cancer cell population after chemotherapy and at metastatic sites. Thus, targeting the vital molecules involved in the maintenance of CSCs enables the elimination of CSCs and improves the outcomes of cancer patients [51,52,53].

Therapies based on the CSC concept are emerging [52]. CD44, the most common CSC marker, could serve as a useful target for CSC immunotherapy [54,55]. Over the past several years, various monoclonal antibodies against CD44 or CD44v isoforms have been evaluated, such as H90 and p245 [56]. H90 reduces leukemic repopulation by targeting acute myelogenous leukemia stem cells [57]. P245 inhibits triple-negative basal-like breast tumor growth in xenograft mice [58]. Moreover, there are several clinical antitumor studies on anti-CD44 antibodies, such as bivatuzumab, which is a humanized monoclonal antibody against CD44v6 [59], and RO5429083, which targets glycosylated CD44 [60]. Because CD44 is highly and functionally expressed in GCSCs, antibody therapy against CD44 may be an effective anticancer tool as a CSC-targeting therapy [61]. In addition, anti-CD44v6 monoclonal antibody-conjugated cold nanostars were developed and can inhibit tumor growth and extend survival in orthotopic and subcutaneous xenograft tumor models of GC in nude mice [62].

In addition, CD133, which has a role as a CSC marker, is a possible therapeutic target in CD133-expressing cancer types [63]. Cytokine-induced killer (CIK) cells bound to an anti-CD3/anti-CD133 bispecific antibody (BsAb) (BsAb-CIKs) are an effector cell population targeting CSCs with high CD133 expression. BsAb-CIK cells have shown strong killing of pancreatic and hepatic cancer cells with high CD133 expression [64]. Recently, a phase I clinical trial investigated the effects of chimeric antigen receptor-modified T cells directed against CD133 (CART-133) on various cancers (hepatocellular carcinoma, pancreatic carcinomas, and colorectal carcinomas). Analysis of biopsied tissues by immunohistochemistry showed that CD133-positive cells were completely eliminated by CART-133 injection [65]. Several kinds of antibodies targeting CD133 have been developed in the past decade. Cytotoxic antibodies against CD133 effectively inhibit the proliferation of cultured GC cells [66]. Anti-CD44 and anti-CD133 antibody-conjugated all-trans retinoic acid-loaded poly (lactide-coglycolide)-lecithin-PEG nanoparticles (CD44/CD133-ATRA-PLPN) were developed to target both CD133-positive and CD44-positive GCSCs. CD44/CD133-ATRA-PLPN show higher inhibition of GCSC growth than single-targeted or nontargeted nanoparticles [67]. Based on the evidence regarding CD44 and CD133, other GCSC markers could be expected to function as therapeutic targets for the elimination of GCSCs.

In addition to CSC markers, CSC-related factors are also noteworthy. The Hippo pathway is a key signaling pathway that regulates organ size through the regulation of cell number and cell size [68]. Deregulation of the hippo pathway can induce tumors in model organisms and is often detected in various human carcinomas [69]. The transcriptional hippo pathway coactivator YAP1 upregulates sox9 expression to endow CSC properties to esophageal cancer cells [70]. Supporting these previous findings, verteporfin, which is a known YAP inhibitor, can inhibit the tumorigenic properties of GCSCs by targeting YAP1/TAZ-TEAD transcriptional activity [71]. In addition to targeting YAP1, verteporfin can downregulate the expression of heat shock protein 90 beta (HSP90) client proteins by blocking clusterin gene expression, resulting in cell death in GCSCs [72].

Hypermethylation of tumor suppressor genes plays a determinative role in carcinogenesis; thus, inhibitors of DNA methylation are taken seriously in molecular therapy [73]. Moreover, the inhibition of DNA methyltransferase (DNMT) can reduce the tumorigenicity of GCSCs [74]. Some potential CSC-related factors are known. For instance, the orphan receptor TR3 (also known as Nur77) can regulate cell proliferation and apoptosis [75,76]. TR3 is an essential factor in the maintenance of stem-like properties in GC cells [77]. Likewise, Sox2 is involved in the determination of cell fate and regulation of embryonic development in cells and can enhance the tumorigenicity and chemoresistance of GCSCs [78]. Given their functional roles in GCSCs, TR3 and Sox2 may be possible therapeutic targets in GCSCs. In this section, we summarized the findings regarding GCSC-targeting therapies. Although evidence is accumulating, further clinical trials are required to prove the clinical significance of GCSC-targeting therapies.

## 5. Conclusions and Future Perspectives

Conventional chemotherapy and molecular targeted therapy in addition to surgical treatment play key roles in prolonging the survival of advanced-stage GC patients. However, clinical outcomes are still disappointing, and these combination therapies are not ideal as a treatment strategy for advanced GCs. The successful determination of CSC markers has allowed the detection of GCSCs, and abundant evidence has shown that GCSCs are often resistant to conventional chemotherapy and closely related to metastasis and recurrence. Moreover, the extensive evidence presented in this review suggests that the tumor microenvironment, especially the immune microenvironment, surrounding GCSCs plays an important role in maintaining the properties of GCSCs. These findings indicate that the CSC niche and niche factors may be potential targets to counteract CSC properties. Therefore, identifying unique molecules and their signaling interactions in the tumor microenvironment through further investigations may lead to the development of an approach complementary to conventional treatment, one that is directed against malignant cells themselves.

## Figures and Tables

**Table 1:** Gastric cancer stem cell markers.

Marker	Significance	References
CD44	Tumorigenicity, spheroid formation, chemoresistance	[6]
CD24/CD44	Tumorigenicity	[7]
CD54/CD44	Tumorigenicity, hierarchical organization	[8]
EpCAM/CD44	Tumorigenicity, phenotypical heterogeneity, chemoresistance	[9]
ALDH1	Tumorigenicity, phenotypical heterogeneity	[10,11]
CD90	Tumorigenicity	[13]
CD71	Tumorigenicity, chemoresistance, tumor cell invasion	[14]
CD133	Poor differentiation, independent prognostic factor	[15,16]
CXCR4	Tumorigenicity, chemoresistance	[17]
Lgr5	Tumorigenicity	[18,19]
